# Histone Deacetylase HDA6 Is Functionally Associated with AS1 in Repression of *KNOX* Genes in *Arabidopsis*

**DOI:** 10.1371/journal.pgen.1003114

**Published:** 2012-12-13

**Authors:** Ming Luo, Chun-Wei Yu, Fang-Fang Chen, Linmao Zhao, Gang Tian, Xuncheng Liu, Yuhai Cui, Jun-Yi Yang, Keqiang Wu

**Affiliations:** 1 Institute of Plant Biology, College of Life Science, National Taiwan University, Taipei, Taiwan; 2 Key Laboratory of Plant Resources, Conservation and Sustainable Utilization, South China Botanical Garden, Chinese Academy of Sciences, Guangzhou, China; 3 University of Chinese Academy of Sciences, Chinese Academy of Sciences, Beijing, China; 4 Southern Crop Protection and Food Research Centre, Agriculture and Agri-Food Canada, London, Ontario, Canada; 5 Institute of Biochemistry, National Chung Hsing University, Taichung, Taiwan; Peking University, China

## Abstract

ASYMMETRIC LEAVES 1 (AS1) is a MYB-type transcription repressor that controls leaf development by regulating *KNOX* gene expression, but the underlying molecular mechanism is still unclear. In this study, we demonstrated that AS1 can interact with the histone deacetylase HDA6 *in vitro* and *in vivo*. The *KNOX* genes were up-regulated and hyperacetylated in the *hda6* mutant, *axe1-5*, indicating that HDA6 may regulate *KNOX* expression through histone deacetylation. Compared with the single mutants, the *as1-1/axe1-5* and *as2-1/axe1-5* double mutants displayed more severe serrated leaf and short petiole phenotypes. In addition, the frequencies of leaf lobes and leaflet-like structures were also increased in *as1-1/axe1-5* and *as2-1/axe1-5* double mutants, suggesting that *HDA6* acts together with *AS1* and *AS2* in regulating leaf development. Chromatin immunoprecipitation assays revealed that HDA6 and AS1 bound directly to *KNAT1*, *KNAT2*, and *KNATM* chromatin. Taken together, these data indicate that HDA6 is a part of the AS1 repressor complex to regulate the *KNOX* expression in leaf development.

## Author Summary

AS1 is a MYB-type transcription repressor that controls leaf patterning by repressing class-1 *KNOX* gene expression. The molecular mechanism by which AS1 represses *KNOX* gene expression is still unclear. In this study, we found that AS1 interacted with the histone deacetylase HDA6. Furthermore, HDA6 repressed *KNOX* gene expression by histone deacetylation. *hda6* mutants displayed serrated leaf and short petiole phenotypes. Additionally, *hda6/as1-1* double-mutant plants showed a more severe phenotype compared to the single mutants, indicating that HDA6 may act together with AS1 in controlling leaf development. Taken together, our data indicated that HDA6 is an important component of the AS1 repressor complex in regulating the *KNOX* gene expression.

## Introduction

The initiation of leaf primordia is established by recruitment of cells from the flanks of the shoot apical meristem (SAM). Meristem activity in the shoot apex is specified in part by the class I *KNOTTED-LIKE HOMOBOX* (*KNOX*) genes [1]–[3]. Lateral organs, such as leaves, are initiated on the flank of SAM, and down-regulation of *KNOX* genes is essential to facilitate this process [1], [4]. Moreover, the silencing of *KNOX* genes is important in developing organs since the ectopic *KNOX* expression during organogenesis resulted in patterning defects and over-proliferation of cells [5]–[7]. Thus, the balance between stem cell differentiation and proliferation that is decisive for plant development is attained, in part through the proper regulation of the *KNOX* expression.

In *Arabidopsis*, the *KNOX* family can be further divided into three classes. Class I *KNOX* genes are similar to *KNOTTED1 (KN1)* in maize, including *BREVIPEDICELLUS (BP)/KNAT1*, *KNAT2*, *KNTA6* and *SHOOTMERISTEMLESS (STM)*. These genes are expressed in the SAM and down-regulated in leaf primordia [8]. Class II *KNOX* genes comprise *KNAT3*, *KNAT4*, *KNAT5* and *KNAT7*, which are broadly expressed. Class III only contains *KNATM*, which is a novel *KNOX* gene lacking the homeodomain. It was demonstrated that KNATM functions together with KNAT1 and BELL proteins by forming heterodimer [9]. Moreover, ectopic expression of *KNATM* resulted in the curled down and serrated rosette leaves in wild type plants [9].

*KNOX* repression is mediated by the orthologous MYB domain proteins ROUGH SHEATH2 (RS2) in maize (*Zea mays*) and ASYMMETRIC LEAVES1 (AS1) in *Arabidopsis thaliana* [10]–[13]. In addition, AS1 interacts with the LATERAL ORGAN BOUNDARIES (LOB) domain protein AS2 and directly represses the expression of *BP/KNAT1* and *KNAT2* [14]–[16]. Previous studies revealed that AS1 and AS2 may recruit a chromatin-remodeling protein Histone Regulatory Homolog 1 (HIRA) to regulate the expression of target genes [17]. Moreover, HIRA has also been shown to interact with a histone deacetylase (HDAC) in animal cells [18].

In this study, we investigated the interaction of AS1 with the histone deacetylase HDA6 and their involvement in leaf development. We demonstrated that HDA6 can interact with AS1 in vivo and in vitro. The *hda6* mutant, *axe1-5*, displayed curling and serrated leaves as well as shorter petioles, suggesting that HDA6 is involved in leaf development. Additionally, HDA6 and AS1 associate directly with the promoters of *KNAT1*, *KNAT2* and *KNATM*. Taken together, our data suggest that HDA6 is a part of the AS1 repression complex to regulate the expression of *KNOX* genes.

## Results

### HDA6 interacts with AS1 in vitro and in vivo

**Figure 1:**
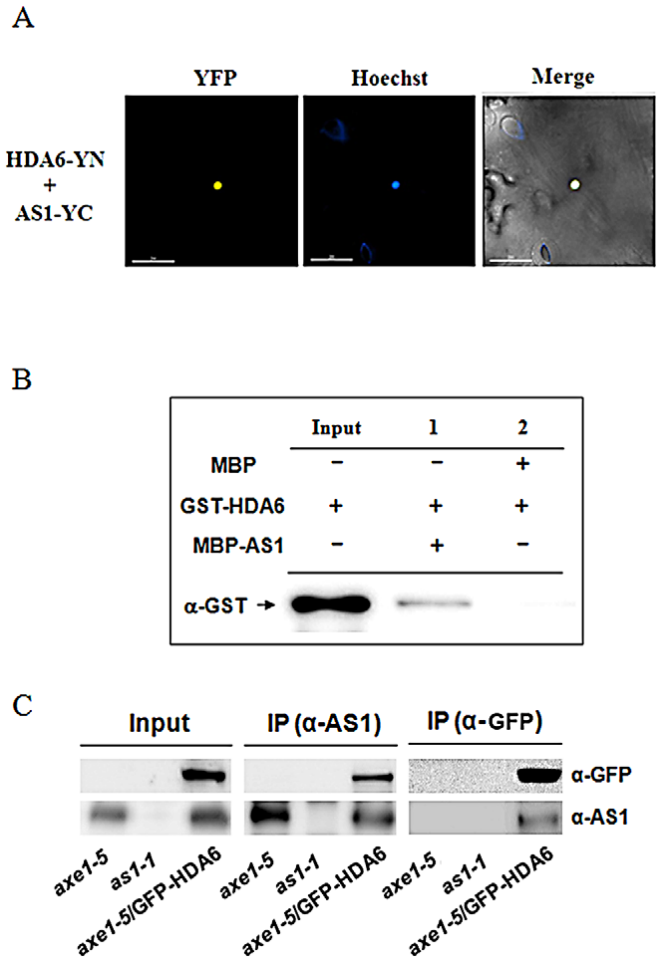
HDA6 interacted with AS1. (A) BiFC in *N. benthamiana* leaves showing interaction between HDA6 and AS1 in living cells. HDA6 and AS1 fused with the N terminus (YN) or C terminus (YC) of YFP were codelivered into tobacco leaves by *Agrobacterium* GV3101 and visualized using a confocal microscope. The nucleus was stained with Hoechst staining (Blue). Bars = 200 µm. (B) HDA6 interacted with AS1 in a pull-down assay. 2 µg MBP-AS1or MBP was incubated with 2 µg GST-HDA6 and MBP affinity resin, and the bound proteins were then eluted from resin and probed with the anti-GST antibody. (C) In vivo interaction between HDA6 and AS1 in *Arabidopsis*. Crude extracts of transgenic plants expressing 35S:GFP-HDA6 in *axe1-5* background were immunoprecipitated (IP) with AS1 or GFP antibody and analyzed by western blotting. *as1-1* and *axe1-5* mutant plants were used as the negative control.

AS1 is a MYB-type transcription repressor that controls leaf patterning by repressing class-1 *KNOX* gene expression [16]. However, the molecular mechanism how AS1 represses *KNOX* gene expression is still unclear. In yeast and mammalian cells, many transcription repressors were found to recruit HDACs to regulate their target genes [19]. To further understand the molecular mechanism of AS1-dependent *KNOX* repression, we analyzed the interaction of AS1 with HDA6, a RPD3-type HDAC in Arabidopsis [20], [21] by using BiFC assays. The coding sequences of HDA6 and AS1 were fused to the N-terminal 174-amino acid portion of yellow fluorescent protein (YFP) in the pEarley-Gate201 vector (pEarleyGate201-YN) or the C-terminal 66-amino acid portion of YFP in the pEarleyGate202 vector (pEarleyGate202-YC) [22]. The *Agrobacterium* cells containing these constructs were co-transfected into *Nicotiana benthamiana* leaves. The yellow fluorescence was observed at the nuclear when HDA6-YN and AS1-YC were transient expressed in *N. benthamiana* leaves, indicating that HDA6 interacted with AS1 *in vivo* (Figure 1A). In contrast, the yellow fluorescence was not observed in the negative controls (Figure S1).

The interaction between HDA6 and AS1 was further confirmed by *in vitro* pull down assays. When purified MBP-AS1 recombinant protein was incubated with glutathione S-transferase (GST)-HDA6 protein, HDA6-GST was pulled down by MBP-AS1 (Figure 1B), indicating that HDA6 was directly associated with AS1. Co-immunoprecipitation (CoIP) assays were also used to analyze the interaction between HDA6 and AS1. A stable transgenic plant expressing *35S:GFP-HDA6* in the *hda6* mutant (*axe1-5*) was generated [23]. Overexpressing *35S:GFP-HDA6* in *axe1-5* complemented the mutant phenotype, suggesting that the GFP-HDA6 fusion protein is functional. Crude extracts (input) of *axe1-5*, *as1-1* and *axe1-5*/*35S:GFP-HDA6* were immunoprecipitated by the AS1 antibody, then analyzed by western blotting. As shown in Figure 1C, GFP-HDA6 was clearly co-immunoprecipitated by endogenous AS1. Furthermore, AS1 protein was also co-immunoprecipitated by GFP-HDA6 when immunoprecipitated by the GFP antibody (Figure 1C). Taken together, our data strongly indicate that HDA6 interacts with AS1 in vitro and in vivo.

### AS1 and AS2 can interact and form homo- and hetero-dimers

**Figure 2:**
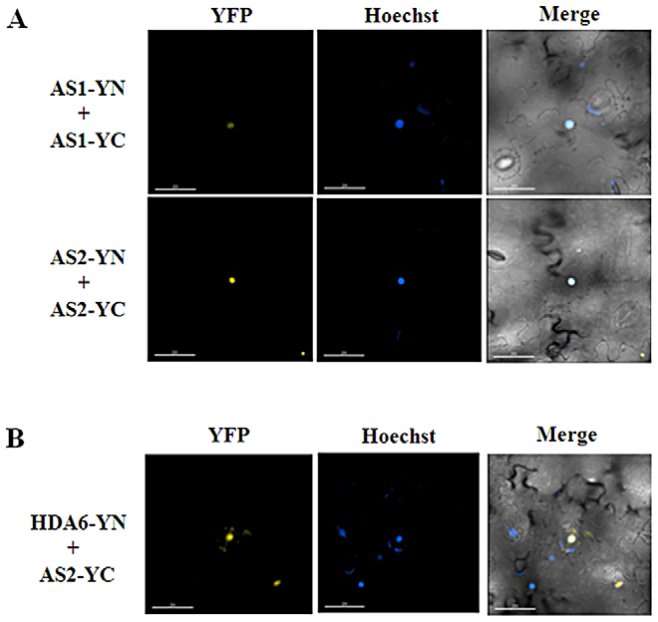
AS1and AS2 formed the homodimer in plants. (A) BiFC in *N. benthamiana* leaves showing the interaction of AS1 and AS2 with itself in living cells. (B) BiFC in *N. benthamiana* leaves showing the interaction between HDA6 and AS2 in living cells. The nucleus was stained with Hoechst staining (Blue). Bars = 200 µm.

Previous studies indicated that AS1 and AS2 can associate together both in yeast cells by yeast two-hybrid assays and in vitro by ELISA experiments using purified His-AS1 and GST-AS2 recombinant proteins [14]. By using BiFC assays, we also found that AS1 and AS2 can interact with each other in *N. benthamiana* leaves (Figure S2). Furthermore, both AS1 and AS2 can also interact with itself (Figure 2A). These observations indicated that AS1 and AS2 can form both homo- and hetero-dimers. The yellow fluorescence was observed at the nucleus when AS1-YN and AS1-YC, AS2-YN and AS2-YC, or AS1-YN and AS2-YC were transient expressed in *N. benthamiana* leaves (Figure 2A and Figure S2). Moreover, the in vivo interaction between HDA6 and AS2 was also found by using BiFC (Figure 2B). Collectively, these results together with the finding that HDA6 interacts with AS1 suggested that HDA6, AS1 and AS2 function together in the same protein complex.

We further tested the protein-protein interactions among HDA6, AS1 and AS2 in the protoplasts isolated from the mutants. By using BiFC assays, we found that HDA6 interacted with AS1 in the nucleus of *as2-1* mutants (Figure S3). Likewise, the interaction of HDA6 and AS2 was also found in the nucleus of *as1-1* mutants. In addition, we also showed that AS1 interacted with AS2 in the nucleus of *axe1-5* mutants. Our data indicate that loss of one component of HDA6, AS1 and AS2 does not affect the interaction of two others in *Arabidopsis*.

### *hda6* mutants displayed curling and serrated leaves

**Figure 3:**
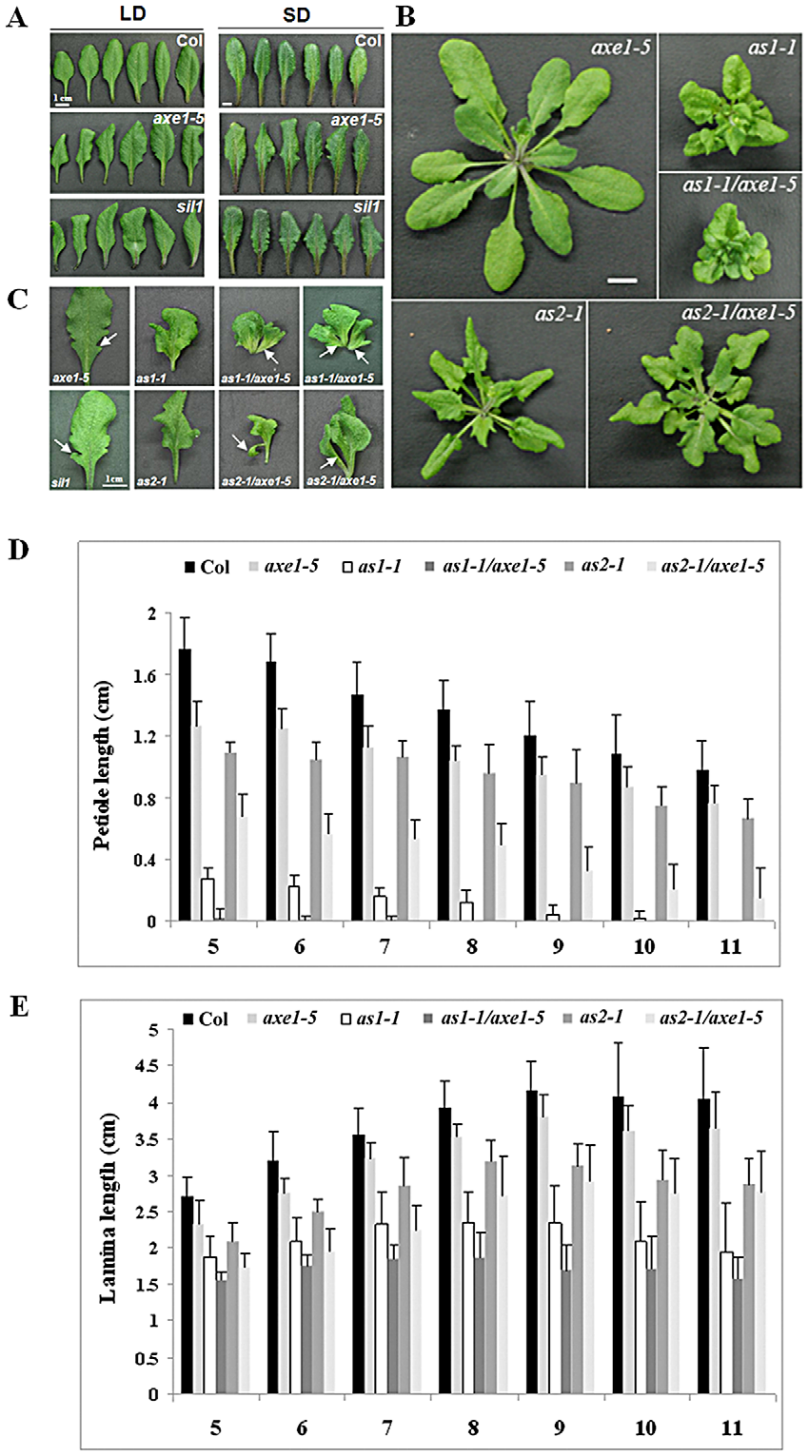
Phenotypes of *axe1-5*, *sil1*, *as1-1*, *as2-1*, *as1-1/axe1-5*, and *as2-1/axe1-5*. (A) The margin serration and curling leaf phenotype of *hda*6 mutants. Col, *axe1-5* and *sil1* plants were grown for 25 days under LD conditions or 75 days under SD conditions. (B) *as1-1/axe1-5* and *as2-1/axe1-5* double mutants displayed a more severe curling and serration leaf phenotype. (C) The phenotype of leaf lobes and leaflet-like structures in *axe1-5*, *as1-1, as2-1, as1-1/axe1-5 and as2-1/axe1-5*. Arrows indicate the leaf lobes in *axe1-5* mutants; or leaflet-like structures in *as1-1/axe1-5* and *as2-1/axe1-5* double mutants. (D) Quantification of the petiole lengths of 5^th^ leaves to 11^th^ leaves. (E) Quantification of the lamina lengths of 5^th^ leaves to 11^th^ leaves. Plants grown under LD conditions for 32 days were used to measure the petiole and lamina lengths. Error bars show SD (n>12).

Previously, we reported that the Arabidopsis *HDA6* is required for flowering time control and the *hda6* mutant, *axe1-5*, displayed a delayed flowering phenotype [23]. In addition, *axe1-5* mutants also displayed the curling leaves under both long-day (LD) and short-day (SD) conditions (Figure 3A). Similar curling and serrated leaves were also found in another *hda6* mutant, *sil1* [25] (Figure 3A), and the *HDA6*-RNAi plants (Figure S4). *hda6* mutants displayed the down curling phenotype on both the distal and lateral axis (Figure 3A). These results demonstrated that HDA6 functions not only in controlling adaxial-abaxial axis, but also in proximal-distal axis and in medial-lateral axis.

### *as1-1/axe1-5* and *as2-1/axe1-5* double mutants displayed a more severe phenotype compared to the single mutants

The *as1* and *as2* mutants of *Arabidopsis thaliana* exhibit pleiotropic phenotypes in leaf development, including the curling and serrated leaves [26]. To examine the genetic interaction between *HDA6* and *AS1* or *AS2*, we generated *as1-1/axe1-5* and *as2-1/axe1-5* double mutants and compared the leaf phenotype of single and double mutants. Under LD conditions, *as1-1/axe1-5* and *as2-1/axe1-5* double mutant plants showed more severe leaf phenotypes compared with *as1-1* and *as2-1* single mutant plants (Figure 3B and 3C).

We also measured the lengths of petioles and lamina in wild type and mutant plants. Compared with wild type, the lengths of the petioles were decreased in *axe1-5* mutants (Figure 3D). *as1-1/axe1-5* and *as2-1/axe1-5* double mutants displayed shorter petioles compared with *as1-1* and *as2-1* single mutant plants (Figure 3B, 3C and Figure 3D). However, the lamina lengths of *as1-1/axe1-5* and *as2-1/axe1-5* did not show significant changes compared with the single mutants (Figure 3E).

We further measured the frequencies of leaf lobe formation in *axe1-5*, *sil1*, *as1-1/axe1-5* and *as2-1/axe1-5* mutants. The frequencies of leaf lobes were significantly increased in *as1-1/axe1-5* and *as2-1/axe1-5* double mutants (Table 1). *as2* mutants produced leaflet-like structures on the petioles [26]. In *as1-1/axe1-5* and *as2-1/axe1-5* double mutants, the frequencies of leaflet-like structures were increased (Table 2), and some of the leaf lobes were similar to leaflet-like structures (Figure 3C). These results suggested that *HDA6* acts with *AS1* and *AS2* in regulating leaf development.

**Table 1:** Frequency of leaf lobes.

		Leaf number							
	n^a^	5	6	7	8	9	10	11	12
*axe1-5*	9	3(33)^b^	6(67)	8(89)	9(100)	8(89)	9(100)	8(89)	8(89)
*Sil1*	6	0(0)	1(17)	2(33)	5(83)	5(83)	5(83)	6(100)	6(100)
*as1-1*	8	7(88)	7(88)	8(100)	8(100)	8(100)	8(100)	8(100)	7(88)
*as1-1/axe1-5*	11	10(91)	11(100)	11(100)	11(100)	11(100)	10(91)	10(91)	11(100)
*as2-1*	12	7(58)	11(92)	10(83)	11(92)	12(100)	12(100)	12(100)	9(75)
*as2-1/axe1-5*	8	8(100)	8(100)	8(100)	8(100)	8(100)	8(100)	8(100)	8(100)

Leaves from 35-day-old plants were examined.

a Number of plants examined.

b Numbers in parentheses show the percentages of leaves on which a lobe was observed.

**Table 2:** Frequency of leaflet-like structure.

		Leaf number								
	n^a^	1	2	3	4	5	6	7	8	9
*as1-1*	8	0(0)^b^	0(0)	0(0)	0(0)	0(0)	0(0)	0(0)	0(0)	0(0)
*as1-1/axe1-5*	11	0(0)	0(0)	0(0)	1(8)	5(46)	4(36)	3(27)	2(18)	8(73)
*as2-1*	12	0(0)	0(0)	0(0)	0(0)	0(0)	1(8)	1(8)	0(0)	4(33)
*as2-1/axe1-5*	8	0(0)	1(13)	0(0)	2(25)	2(25)	2(25)	0(0)	0(0)	4(50)

Leaves from 35-day-old plants were examined.

a Number of plants examined.

b Numbers in parentheses show the percentages of leaves on which the leaflet-like structure was observed.

### Expression of *KNOX* genes was increased in *axe1-5*, *as1-1*, and *as2-1* mutant plants

**Figure 4:**
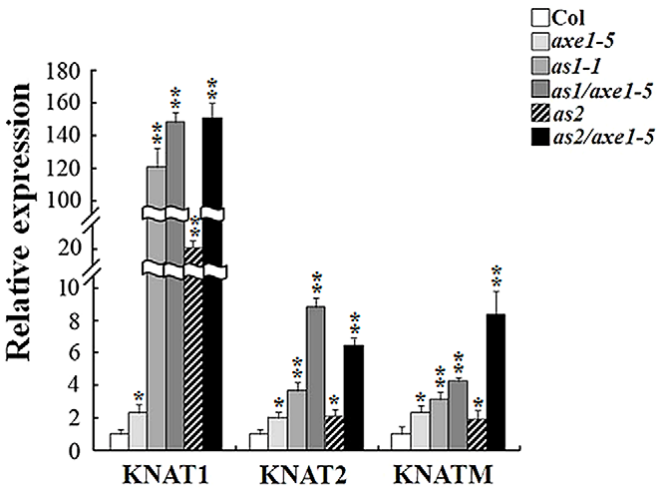
Expression of *KNOX* genes was increased in *axe1-5*, *as1-1*, *as2-1*, *as1-1/axe1-5*, and *as2-1/axe1-5* mutant plants. qRT-PCR analysis of gene expression of *KNAT1*, *KNAT2* and *KNATM* in *axe1-5*, *as1-1*, *as2-1*, *as1-1/axe1-5* and *as2-1/axe1-5* plants grown under LD conditions for 20 days. Asterisks mark values that are significantly different from the Col wild type (t- test, **P<0.01, *P<0.05).

We further analyzed the gene expression by quantitative reverse transcription (qRT)-PCR in mutant plants. Compared with Col wild type, no significant changes were found in the expression of *AS1* and *AS2* in the *axe1-5* (Figure S5). As shown in Figure 4, the expression of *KNAT1*, *KNAT2* and *KNATM* was increased in *axe1-5* compared to Col wild type. Consistent with the previous study [13], the transcript levels of *KNAT1* and *KNAT2* were elevated in *as1-1* and *as2-1* mutant plants. In addition, the expression of *KNATM* was also up-regulated in *as1-1* and *as2-1* mutant plants. Moreover, the expression of *KNAT1*, *KNAT2* and *KNATM* was highly increased in *as1-1/axe1-5* and *as2-1/axe1-5* double mutants compared with their corresponding single mutants. These data indicate that *HDA6* may function synergistically with *AS1* and *AS2* in regulating the expression of *KNOX* genes.

We also analyzed the expression of *PHB*, *PHV*, *CUC1* and *CUC2*, which were involved in leaf development through the miRNA regulated pathway [27]–[30]. However, no significant different was found in the expression of *PHB*, *PHV*, *CUC1* and *CUC2* (Figure S5).

### Histone H3 acetylation levels of *KNOX* genes were increased in *axe1-5*, *as1-1/axe1-5*, and *as2-1/axe1-5* mutant plants

**Figure 5:**
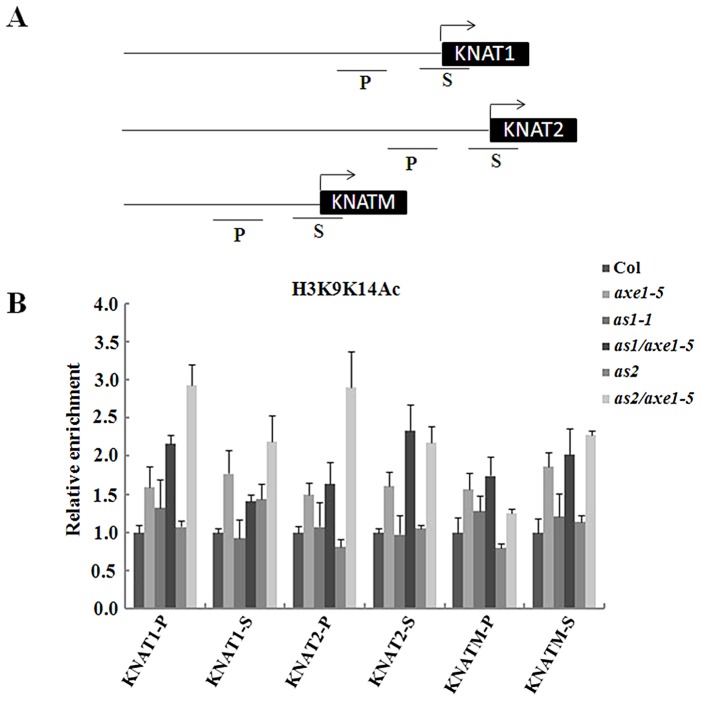
Levels of H3K9K14Ac in *KNAT1*, *KNAT2*, and *KNATM* chromatin in *as1-1/axe1-5* and *as2-1/axe1-5* double mutants. (A) Schematic diagram of *KNAT1*, *KNAT2*, *KNATM* promoter and transcription start regions examined by ChIP. P, promoter region; S, transcription start region. (B) Relative levels of H3K9K14Ac in Col and mutant plants. The amount of DNA after ChIP was quantified and normalized to an internal control (*ACTIN2*). The values shown are means ± SD.

To determine whether the high expression of *KNOX* genes in the mutants is related to histone hyperacetylation in chromatin, ChIP assays were used to analyze the histone H3 acetylation levels of *KNAT1*, *KNAT2* and *KNATM*. The relative enrichment of histone H3 acetylation was determined by real-time PCR using primers specific for the proximal promoter (within 500 bp upstream of the transcription starting sites) and transcription start regions of individual genes. As shown in Figure 5, levels of histone H3 acetylation were slight elevated in the proximal promoter and transcription start regions of *KNAT1*, *KNAT2* and *KNATM* in *axe1-5*, suggesting that HDA6 may regulate these genes expression by chromatin deacetylation. We further analyzed histone acetylation levels of *KNAT1*, *KNAT2* and *KNATM* in *as1-1*, *as2-1* and the double mutants. As shown in Figure 5B, hyperacetylation of histone H3 was found in the promoter and first exon of *KNAT1*, *KNAT2* and *KNATM* in *as1-1/axe1-5* and *as2-1/axe1-5* double mutants. In contrast, hyperacetylation of histone H3 was not found in *as1-1* and *as2-1* single mutants. These results suggested that hyperacetylation of histone H3 in *KNAT1*, *KNAT2* and *KNATM* found in *as1-1/axe1-5* and *as2-1/axe1-5* double mutants was caused by the *hda6* mutation.

Histone H3K4Me3 is another chromatin mark associated with active genes. We also investigated the histone H3K4Me3 level in *axe1-5* mutants. However, no significant changes in the H3K4Me3 of *KNAT1*, *KNAT2* and *KNATM* were found (Figure S6A). H3K9Me2 was reported as a chromatin marker associated with gene repression. No significant changes in the level of histone H3K9Me2 was found in *axe1-5* mutants (Figure S6B).

### HDA6 and AS1 bound to *KNAT1*, *KNAT2*, and *KNATM* chromatin

**Figure 6:**
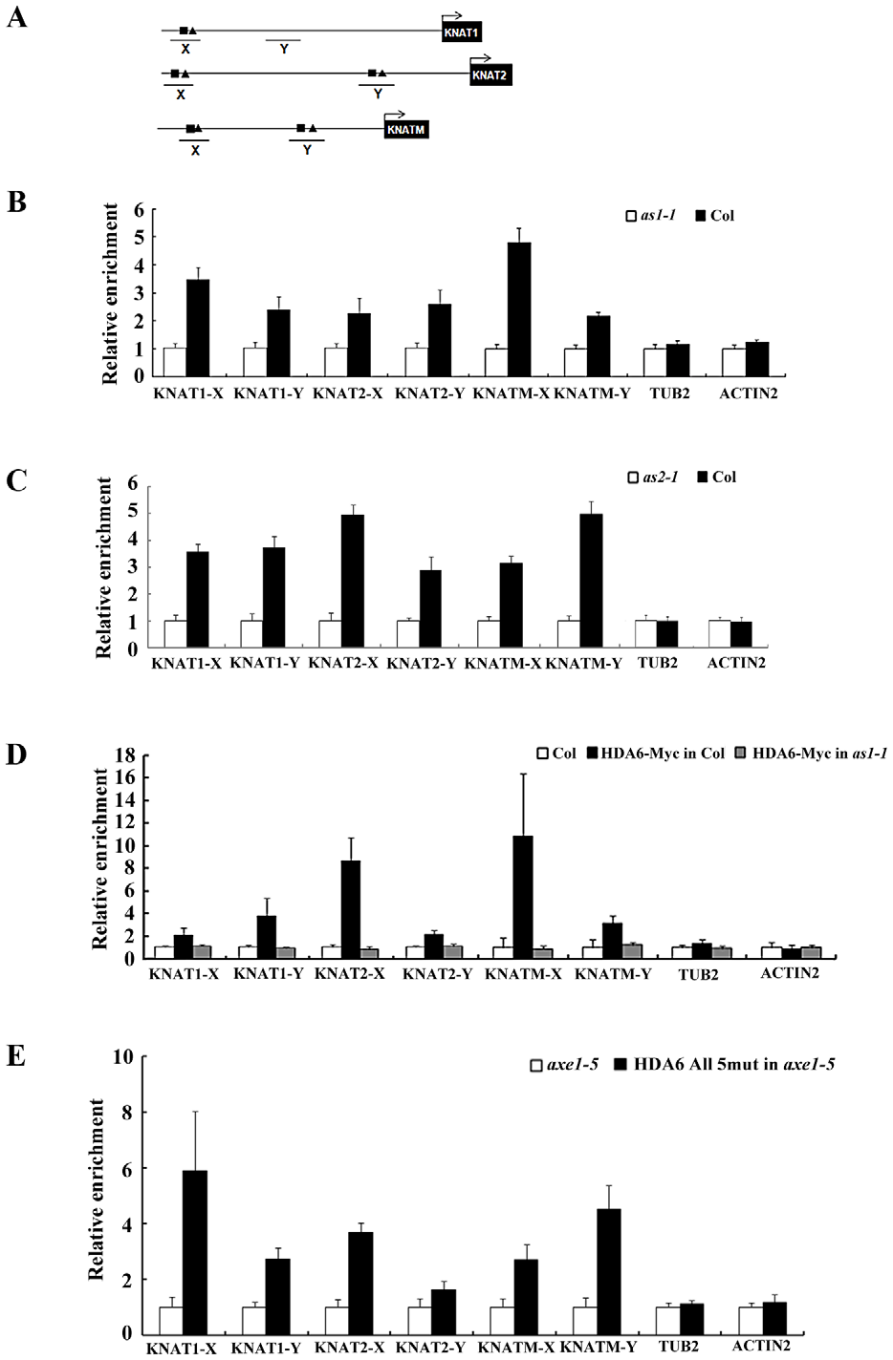
AS1 and HDA6 bound to *KNAT1*, *KNAT2*, and *KNATM* promoters. (A) Diagrams of *KNAT1*, *KNAT2*, *KNATM* and the regions examined by ChIP. X and Y indicate regions examined by ChIP. Motif I and II are indicated by square and triangle, respectively. (B, C) Recruitment of AS1 to the *KNAT1*, *KNAT2* and *KNATM* loci. Relative fold enrichment was calculated by dividing the amount of DNA immunoprecipitated from Col plants by that from the *as1-1* or *as2-1* plants and compared with input DNA. Plants were grown under LD conditions for 18 days. *ACTIN2* and *TUB2* were used as the negative controls. The values shown are means ± SD. (D) Recruitment of HDA6 to the *KNAT1*, *KNAT2* and *KNATM* loci. Relative fold enrichment was calculated by dividing the amount of DNA immunoprecipitated with the anti-Myc antibody from Col plants by that from the *HDA6*-Myc transgenic plants and compared with input DNA. HDA6-Myc in Col is a transgenic line expressing a Myc-tagged HDA6 in Col background, whereas HDA6-Myc in *as1-1* is a transgenic line expressing a Myc-tagged HDA6 in *as1-1* mutants. (E) Recruitment of HDA6 to the *KNAT1*, *KNAT2* and *KNATM* is independent of its catalytic activity. DNA fragments coimmunoprecipitated with the anti-Flag antibody relative to *axe1-5* were analyzed by ChIP. HDA6 All 5 mut in *axe1-5* is a transgenic line expressing HDA6 bearing the five amino acid mutation of the active site in *axe1-5* mutants. Plants were grown under LD conditions for 18 days. *ACTIN2* and *TUB2* were used as the negative controls. The values shown are means ± SD.

The direct association between AS1 and HDA6 suggested that AS1 may recruit HDA6 to repress the downstream target genes. Previous studies demonstrated that the AS1 repressor complex binds directly to the regulatory motif I (CWGTTD) and motif II (KMKTTGAHW) on the promoters of the *KNAT1* and *KNAT2* [16]. We also found the conserved motif I and motif II in two promoter regions (KNAMT-X and KNAMT-Y) of *KNATM* (Figure 6A and Figure S7). To investigate whether AS1 binds directly to *KNAT1*, *KNAT2* and *KNATM*, ChIP analyses using the AS1 antibody were performed in Col wild type and *as1-1* mutants. Consistent with the previous report [16], AS1 can bind to the promoters of *KNAT1* and *KNAT2* (Figure 6B). In addition, AS1 can also bind directly to *KNATM* (Figure 6B). In comparison, AS1 cannot bind to the control genes, *ACTIN2* and *TUB2*. To analyze whether the binding of AS1 to *KNAT1*, *KNAT2* and *KNATM* requires the presence of AS2, we also performed ChIP assays using the *as2-1* mutants. We found the loss of binding of AS1 to the *KNOX* chromatin in the *as2-1* mutant (Figure 6C), suggesting that AS2 is required for the binding of AS1 to the *KNOX* genes.

To examine whether HDA6 can binds directly to *KNAT1*, *KNAT2* and *KNATM*., transgenic plants expressing HDA6-Myc were subjected to ChIP analysis using an anti-Myc antibody. As shown in Figure 6D, ChIP analyses revealed that HDA6 can bind to the promoters of *KNAT1*, *KNAT2* and *KNATM*. We also analyze whether HDA6 recruitment is dependent on AS1. ChiP assays were performed using an anti-Myc antibody in transgenic plants expressing the HDA6-Myc in *as1* mutants. As shown in Figure 6D, HDA6 cannot bind to *KNAT1*, *KNAT2* and *KNATM* in *as1* mutants, suggesting that AS1 is required to recruit HDA6.

To analyze whether the HDA6 binding is dependent on its catalytic activity, we performed ChIP assays using an anti-FLAG antibody in transgenic plants (HDA6 all 5 mut in *axe1-5*) expressing the FLAG-tagged HDA6 bearing the five amino acid mutation of the active site in *axe1-5* mutants [31]. As show in Figure 6E, the active site mutant HDA6 can still bind to *KNAT1*, *KNAT2* and *KNATM*, suggesting that HDA6 recruitment is independent of its catalytic activity. Taken together, our findings suggested that HDA6, AS1 and AS2 act together and directly repress the expression of *KNOX* genes in *Arabidopsis*.

## Discussion

### HDA6 regulates the *KNOX* expression

The *Arabidopsis* genome sequence contains 9 *KNOX* genes, which can be further classified into 3 classes [32]. In leaves, AS1 and AS2 down-regulate class I *KNOX* genes, but not *STM*; conversely, STM represses *AS1* expression in the SAM [12], [33]. Downregulation of *KNOX* genes expression is a vital step in leaf initiation, and silencing of these genes needs to be maintained for normal organogenesis [13], [15]. In this study, we demonstrated that *hda6* mutants displayed the curling and serrated leaves and shorter petioles. Compared with the single mutants, *as1-1/axe1-5* and *as2-1/axe1-5* double mutants show more severer phenotypes on curling leaves, petiole lengths, and leaflet-like structures, supporting that HDA6 acts synergistically with AS1 and AS2 in the regulation of leaf development.

*KNAT1* and *KNAT2* were previously found to be repressed by AS1 and AS2 [14]–[16]. Our results indicated that the transcript levels of *KNAT1*, *KNAT2* and *KNATM* were altered in *axe1-5*, *as1-1* and *as2-1* mutants. Furthermore, the expression of *KNAT1*, *KNAT2* and *KNATM* was highly increased in *as1-1/axe1-5* and *as2-1/axe1-5* double mutants compared to their corresponding single mutants. In addition, levels of histone H3 acetylation was elevated in *KNAT1*, *KNAT2* and *KNATM* loci in *axe1-5*, *as1-1/axe1-5* and *as2-1/axe1-5* mutants, suggesting that HDA6 is required for the repression of *KNOX* genes by chromatin deacetylation. ChIP analyses revealed that HDA6 and AS1 bound directly to the promoters of *KNAT1*, *KNAT2* and *KNATM*. These data indicate that HDA6 and AS1 function together in controlling *KNOX* gene expression through histone dacetylation. In addition, AS1 is required to recruit HDA6 in *KNOX* repression HDA6 cannot bind to *KNAT1*, *KNAT2* and *KNATM* in *as1* mutants, suggesting that AS1 is required to recruit HDA6 in *KNOX* repression.

Microarray gene expression analyses revealed that a large number of loci are differently expressed in *hda6* mutants [23], [34], indicating that HDA6 may play multiple roles in different development processes. Recent studies suggested that the expression of *KNOX* genes is only one important factor for leaf development [28], [30]. Further analysis is required to determine whether HDA6 is involved in other leaf development pathways.

### *KNATM* is a novel target of the AS1–AS2 complex

*AS1* is a Myb domain transcription factor related to *RS2* in maize and *PHANTASTICA* in *Antirrhinum* [12]. Mutations in *AS1* result in abnormal leaves, with marginal outgrowths or lobes [12], [13], [33], [35] *AS2* encodes a LOB domain protein containing a leucine-zipper motif [36]–[38]. Mutations in the *as2* gene cause a phenotype similar to *as1* mutants [13], [33]. Previous studies indicated that AS1 and AS2 can associate together both in vitro and in yeast cells [14]. By using the BiFC assay, we found that AS1 and AS2 can interact and form homo and hetero-dimer in plant cells. These data suggested that AS1 and AS2 function in the same protein complex.

A recent study indicated that AS1 functions as a transcriptional repressor and binds directly to its *KNOX* targets when in a complex with AS2 [16]. It was found that the AS1–AS2 repressor complex binds directly to the regulatory motif I (CWGTTD) and motif II (KMKTTGAHW) in the promoters of the *KNAT1* and *KNAT2* [16]. Similar to *KNAT1* and *KNAT2*, we also found the conserved motif I and motif II in the promoter of *KNATM*. *KNATM* is a novel *Arabidopsis* Class III *KNOX* gene that has a MEINOX domain but lacks the homeodomain [9]. ChIP assayes revealed that AS1 can bind directly to the promoter regions of *KNAT1*, *KNAT2* and *KNATM*. These data suggested that in addition to *KNAT1* and *KNAT2*, the AS1–AS2 complex is also targeted to *KNATM* by binding to the conserved motifs I and II. To our knowledge, this is the first study demonstrating that *KNATM* is regulated by AS1 and *AS2*.

### HDA6 is one of the epigenetic components involved in the AS1–AS2–mediated *KNOX* repression

Recent studies suggested that the AS1–AS2 complex binds to the *KNAT1* and *KNAT2* promoters and recruit the chromatin-remodeling protein HIRA to maintain the chromatin in a stable repressive state [15], [16], [39]. In mammalian cells, HIRA was shown to interact with a histone deacetylase [18]. Moreover, it was observed that Arabidopsis seedlings treated with TSA, an inhibitor of HDACs, produced abaxialized filamentous leaves, indicating the involvement of HDACs in leaf morphogenesis [24]. In this study, we provided direct evidence indicating that HDA6 is involved in leaf morphogenesis by interacting with AS1 and AS2 to regulate the *KNOX* expression. Compared with the single mutants, *as1-1/axe1-5* and *as2-1/axe1-5* double mutants show more severe phenotypes on curling leaves, petiole lengths, and leaflet-like structures, supporting that HDA6 acts with AS1 and AS2 to regulate leaf development. Taken together, our results demonstrated that histone deacetylation is one of the epigenetic components involved in AS1–AS2 complex-mediated *KNOX* repression. HDA6 may therefore be part of the AS1–AS2 repression complex to repress the target gene expression. Our data indicate that loss of one component of HDA6, AS1 and AS2 does not affect the interaction of two others in *Arabidopsis*. Previous studies indicated that the interaction between AS1 and AS2 is required for their binding to the promoters of *KNOX* genes, because neither AS1 nor AS2 alone was able to bind to the target DNA sequences in vitro [40]. We observed the loss of binding of AS1 to the *KNOX* chromatin in the *as2-1* mutant, suggesting that AS2 is required for the AS1 binding. Furthermore, HDA6 cannot bind to *KNOX* chromatin in *as1-1* mutants, indicating that AS1 is required to recruit HDA6. Taken together, both AS1 and AS2 are required for the recruitment of HDA6 to chromatin in repression of *KNOX* genes.

A recent work has also shown that the Polycomb Repressive Complexes (PRCs) repress *KNOX* transcription [40]. It was found that CLF-containing PRC2 regulates *KNOX* genes by trimethylation of histone H3K27 [41]. Thus, AS1 and AS2 may also recruit other chromatin factors such as PRCs to regulate class I *KNOX* genes. Taken together, our results suggested that HDA6 is one of the epigenetic components involved in the AS1–AS2 complex-mediated *KNOX* repression during leaf development in *Arabidopsis*.

## Materials and Methods

### Plant materials

*Arabidopsis thaliana* was grown in 23°C under LD (16 h light/8 h dark) or SD (8 h light/16 h dark) conditions. *axe1-5*, *sil1*, *as1-1* and *as2-1* are in the Col background, whereas the *HDA6* RNAi lines CS24038 and CS24039 are in Ws background.

### Quantitative RT–PCR analysis

Arabidopsis leaves (0.2 g) were ground with liquid nitrogen in a mortar and pestle and mixed with 1 ml Trizol Reagent (Invitrogen) to isolate total RNA. After treated with DNase (Promega), two microgram of total RNA was used for the first-strand cDNA synthesis. cDNA was synthesized in a volume of 20 µl that contained the Moloney Murine Leukemia Virus Reverse Transcriptase buffer (Promega), 1.5 µM poly(dT) primer, 0.5 mM deoxyribonucleotide triphosphates, 25 units RNasin ribonuclease inhibitor, and 200 units Moloney Murine Leukemia Virus Reverse Transcriptase at 37°C for 1 h.

cDNAs obtained from reverse transcription were used as a template to run real-time PCR. The following components were added to a reaction tube: 9 µL of iQ SYBR Green Supermix solution (Bio-Rad), 1 µL of 5 µM specific primers, and 8 µL of the diluted cDNA template. Thermocycling conditions were 95°C for 3 minutes followed by 40 cycles of 95°C for 30 s, 60°C for 30 s, and 72°C for 20 s, with a melting curve detected at 95°C for 1 minute, 55°C for 1 minute, and detected the denature time from 55°C to 95°C. Each sample was quantified at least triplicate and normalized using *Ubiquitin 10* as an internal control. The gene-specific primer pairs for quantitative RT-PCR are listed in Table S1.

### ChIP assays

ChIP assay was carried out as described [42]. Chromatin extracts were prepared from 10 day old seedlings treated with formaldehyde. The chromatin was sheared to an average length of 500 bp by sonication and immunoprecipitated with specific antibodies including anti-acetylated histone H3K9K14 (Catalogue no. 06-599, Millipore), anti-trimethylated histone H3K4 (Catalogue no. 04-745, Millipore), anti-c-Myc (Catalogue no. M4439, Sigma) and anti-FLAG (Catalogue no. F1804, Sigma). The DNA cross-linked to immunoprecipitated proteins was analyzed by real-time PCR. Relative enrichments of various regions of *KNAT1*, *KNAT2* and *KNATM* in *axe1-5*, *as1-1* and *as1-1*/*axe1-5* over Col were calculated after normalization to *ACTIN2*. Each of the immunoprecipitations was replicated three times, and each sample was quantified at least in triplicate. The primers used for real-time PCR analysis in ChIP assays are listed in Table S2.

### BiFC assays

To generate the constructs for BiFC, full-length coding sequences of *HDA6*, *AS1* and *AS2* were PCR-amplified using Pfu polymerase (Finnzymes). The PCR products were subcloned into the pENTR/SD/D-TOPO or pCR8/GW/TOPO vector and then recombinated into the pEarleyGate201-YN and pEarleyGate202-YC vectors [22]. The resulting constructs were transformed into the *Agrobacterium* GV3101 and the Agrobacteria containing these constructs were cotransfected into five week old *Nicotiana benthamiana* leaves. For the protoplast transient expression, HDA6, AS1 and AS2 fused with pEarleyGate201-YN or pEarleyGate201-YC were co-transfected into protoplasts by PEG transfection [43]. Transfected leaves and protoplasts were imaged using TCS SP5 (Leica) Confocal Spectral Microscope Imaging System.

### In vitro pull-down assays

Pull-down assays were performed as previously described [44] with some modifications. 2 µg Myelin basic protein (MBP) and MBP-AS1 recombinant proteins were incubated with 30 µl of MBP resin in a total volume of 500 µl of MBP binding buffer (20 mM Tris-HCl, pH 7.5, 200 mM NaCl, 1 mM EDTA) for 2 h at 4°C, and the binding reaction was washed 3 times by the binding buffer, then 2 µg GST-HDA6 recombinant protein was added and incubated for additional 2 h at 4°C. After extensive washing (at least 8 times), the pulled down proteins were eluted by boiling, separated by 10% SDS-PAGE, and detected by western blotting using an anti-GST antibody.

### Coimmunoprecipitation assays

Coimmunocipitation assays were performed as previous described [23]. The 20-day-old *axe1-5/35S:GFP-HDA6*, *axe1-5* and *as1-1* plants were harvested and ground in liquid nitrogen. Total proteins were extracted in an extraction buffer (50 mM Tris-HCl, pH 7.4, 150 mM NaCl, 2 mM MgCl_2_, 1 mM DTT, 20% glycerol, and 1% CA-630) containing protease inhibitor cocktail (Roche). Cell debris was pelleted by centrifugation at 14,000 g for 30 min. The supernatant was incubated with anti-AS1 or anti-GFP specific antibody overnight at 4°C by gently rotation, then 50 µl of protein G agarose beads (Millipore) was added. After 3 h of incubation at 4°C by gently rotation, the beads were centrifuged and washed five times with a washing buffer (50 mM Tris-HCl, pH 7.4, 150 mM NaCl, 2 mM MgCl_2_, 1 mM DTT, 10% glycerol, and 1% CA-630). Proteins were eluted with 40 µl of 2.5× sample buffer and analyzed by western blotting using anti-AS1 and anti-GFP (Santa Cruz Biotechnologies) antibodies.

## Supporting Information

Figure S1 Negative controls of BiFC in *N. benthamiana* leaves. HDA6, AS1 and AS2 fused with YN or YC and the empty vector (YN and YC) were co-delivered into tobacco leaves as negative controls. No YFP signals were detected. The nucleus was stained with Hoechst nuclear stain (Blue).
(TIF)

Figure S2 AS1 and AS2 formed the heterodimer in plants. BiFC in *N. benthamiana* leaves showing interaction between AS1 and AS2 in living cells. Arrows indicate nuclear fluorescence.
(TIF)

Figure S3 Interaction among HDA6, AS1 and AS2 in the protoplasts of wild-type and mutants in Arabidopsis. (A) BiFC showing interaction between HDA6 and AS1 in wild type Col and *as2-1* mutant plants. (B) BiFC showing interaction between HDA6 and AS2 in wild type Col and *axe1-5* mutant plants. (C) BiFC showing interaction between AS1 and AS2 in wild type Col and *axe1-5* mutant plants. (D) Negative controls of BiFC. HDA6, AS1 and AS2 fused with N-terminal (pEarleyGate201-YN) or C-terminal (pEarleyGate201-YC) were co-transformed into protoplasts of wild-type Col and mutants. VirD2NLS fused with mCherry was used as a nuclear marker (Blue).
(TIF)

Figure S4 Leaf phenotype of *HDA6*-RNAi plants. Ws and *HDA6*-RNAi (CS24038 and CS24039) plants were grown under SD conditions for 30 days. Both CS24038 and CS24039 plants displayed the margin serration and curling leaf phenotypes.
(TIF)

Figure S5 The expression of *AS1*, *AS2*, *PHB*, *PHV*, *CUC1*, and *CUC2* in *axe1-5* mutants. qRT-PCR analyses of gene expression in *axe1-5* plants grown under LD conditions for 20 days. The values shown are means ± SD.
(TIF)

Figure S6 Levels of H3K4Me3 and H3K9Me2 in *KNAT1*, *KNAT2* and *KNATM* in *axe1-5* mutants. Relative levels of H3K4Me3 (A) and H3K9Me2 (B) in Col and *axe1-5* mutant plants. P, promoter region; S, transcription start region. The amount of DNA after ChIP was quantified and normalized to an internal control (*ACTIN2* or *TA3*). The values shown are means ± SD.
(TIF)

Figure S7 Sequences of motif I and motif II in *KNAT1*, *KNAT2* and *KNATM* promoters. Inferred consensus sequences for the AS1 binding motifs and their positions relative to the translation start codon of *KNAT1*, *KNAT2* and *KNATM* are also shown.
(TIF)

Table S1 Gene-specific primer pairs for quantitative RT–PCR.
(DOC)

Table S2 Primers used for quantitative RT–PCR analyses in ChIP assays.
(DOC)
