# Supplementary figures and images for: Histone Deacetylase HDA6 Is Functionally Associated with AS1 in Repression of KNOX Genes in Arabidopsis
Source: PLoS Genet. 2012 Dec 13;8(12):e1003114. doi: 10.1371/journal.pgen.1003114 (PMC3521718; doi:10.1371/journal.pgen.1003114)

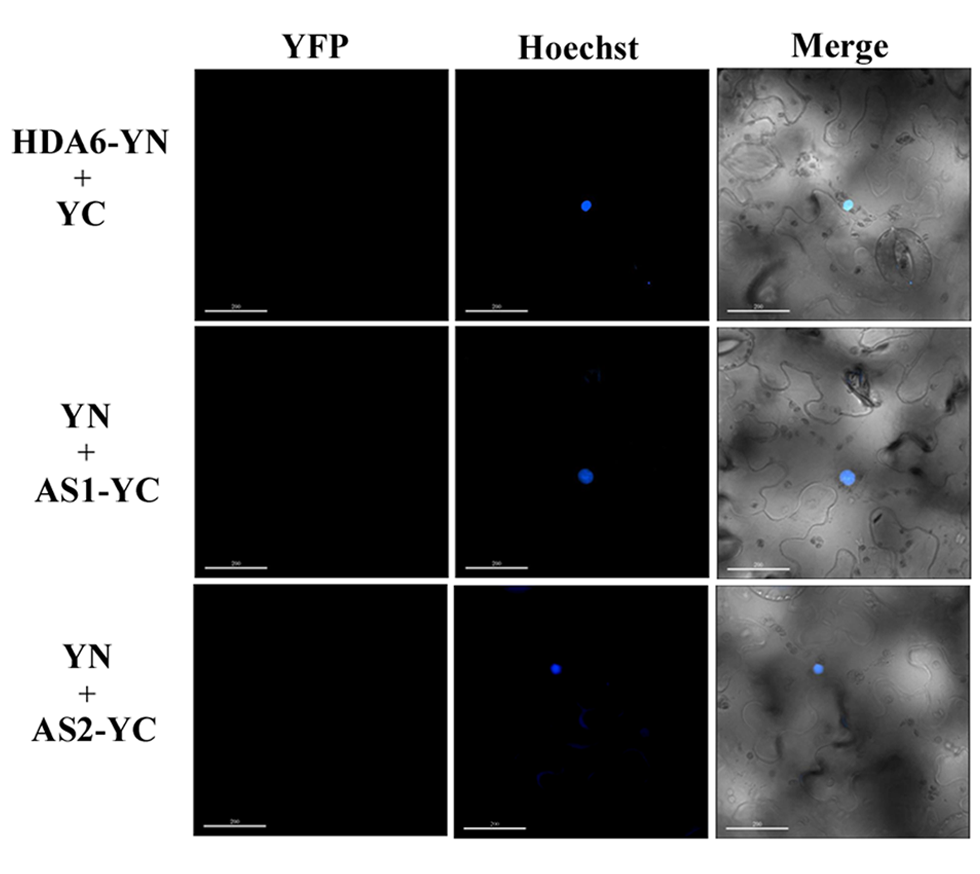

Supplement: Figure S1 — Negative controls of BiFC in N. benthamiana leaves. HDA6, AS1 and AS2 fused with YN or YC and the empty vector (YN and YC) were co-delivered into tobacco leaves as negative controls. No YFP signals were detected. The nucleus was stained with Hoechst nuclear stain (Blue). (TIF) [file pgen.1003114.s001.tif]

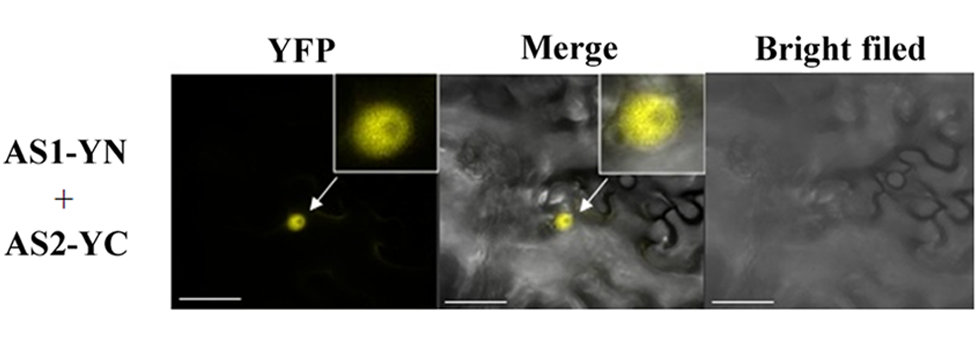

Supplement: Figure S2 — AS1 and AS2 formed the heterodimer in plants. BiFC in N. benthamiana leaves showing interaction between AS1 and AS2 in living cells. Arrows indicate nuclear fluorescence. (TIF) [file pgen.1003114.s002.tif]

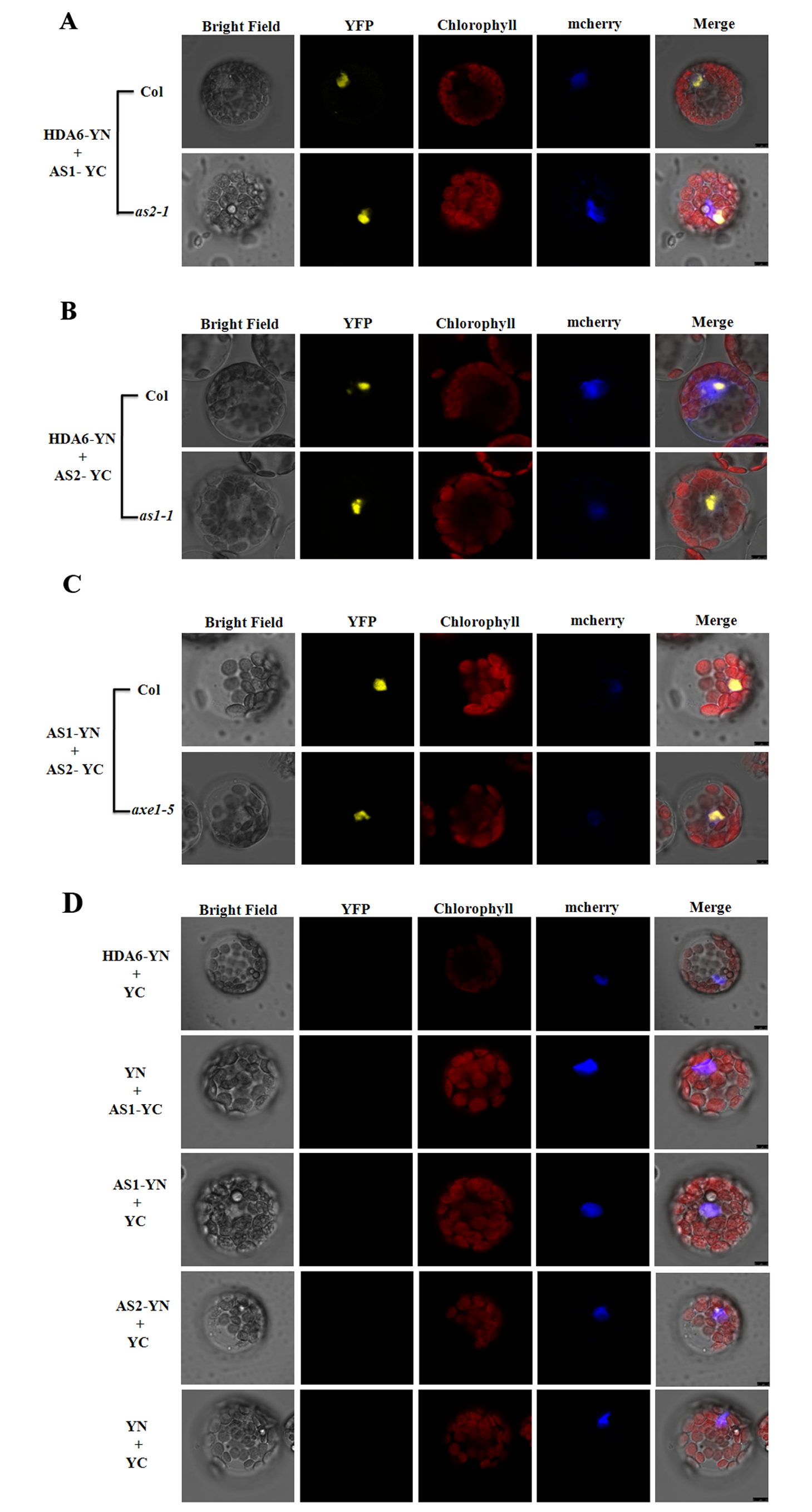

Supplement: Figure S3 — Interaction among HDA6, AS1 and AS2 in the protoplasts of wild-type and mutants in Arabidopsis. (A) BiFC showing interaction between HDA6 and AS1 in wild type Col and as2-1 mutant plants. (B) BiFC showing interaction between HDA6 and AS2 in wild type Col and axe1-5 mutant plants. (C) BiFC showing interaction between AS1 and AS2 in wild type Col and axe1-5 mutant plants. (D) Negative controls of BiFC. HDA6, AS1 and AS2 fused with N-terminal (pEarleyGate201-YN) or C-terminal (pEarleyGate201-YC) were co-transformed into protoplasts of wild-type Col and mutants. VirD2NLS fused with mCherry was used as a nuclear marker (Blue). (TIF) [file pgen.1003114.s003.tif]

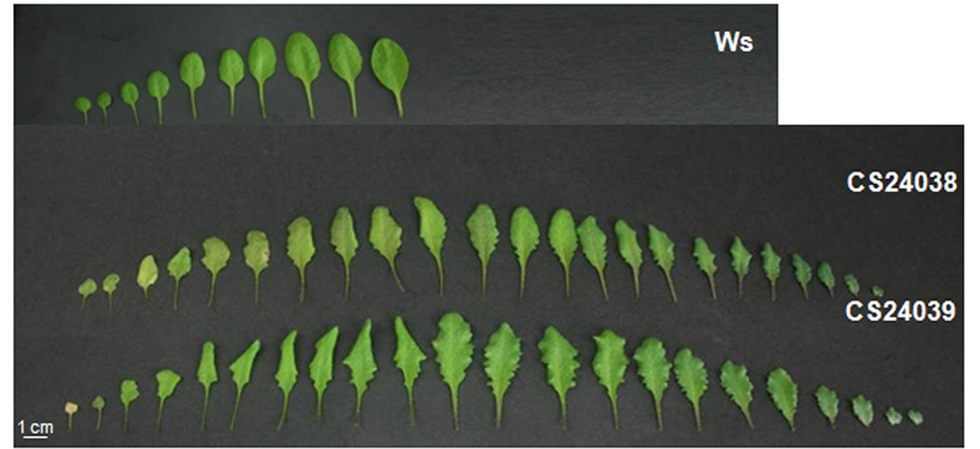

Supplement: Figure S4 — Leaf phenotype of HDA6-RNAi plants. Ws and HDA6-RNAi (CS24038 and CS24039) plants were grown under SD conditions for 30 days. Both CS24038 and CS24039 plants displayed the margin serration and curling leaf phenotypes. (TIF) [file pgen.1003114.s004.tif]

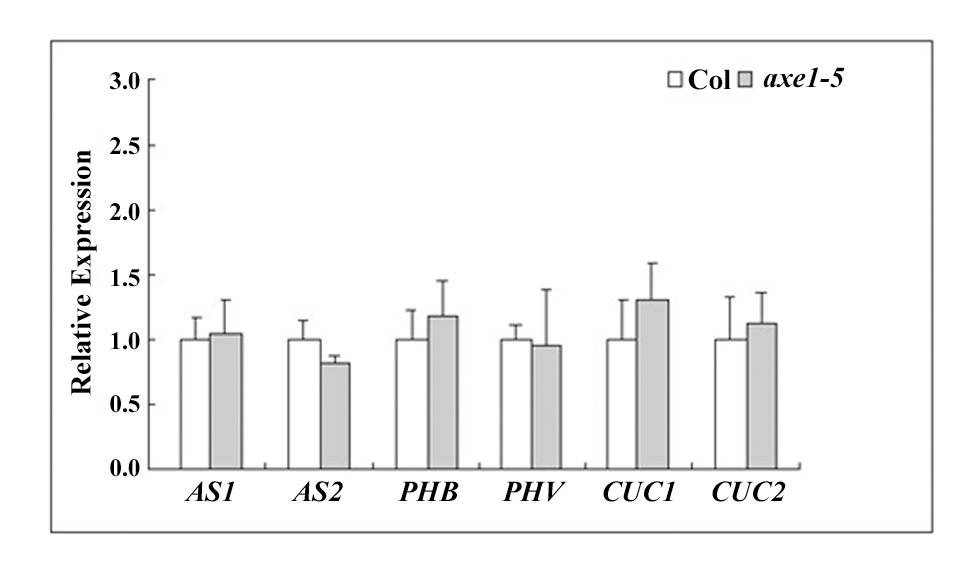

Supplement: Figure S5 — The expression of AS1, AS2, PHB, PHV, CUC1, and CUC2 in axe1-5 mutants. qRT-PCR analyses of gene expression in axe1-5 plants grown under LD conditions for 20 days. The values shown are means ± SD. (TIF) [file pgen.1003114.s005.tif]

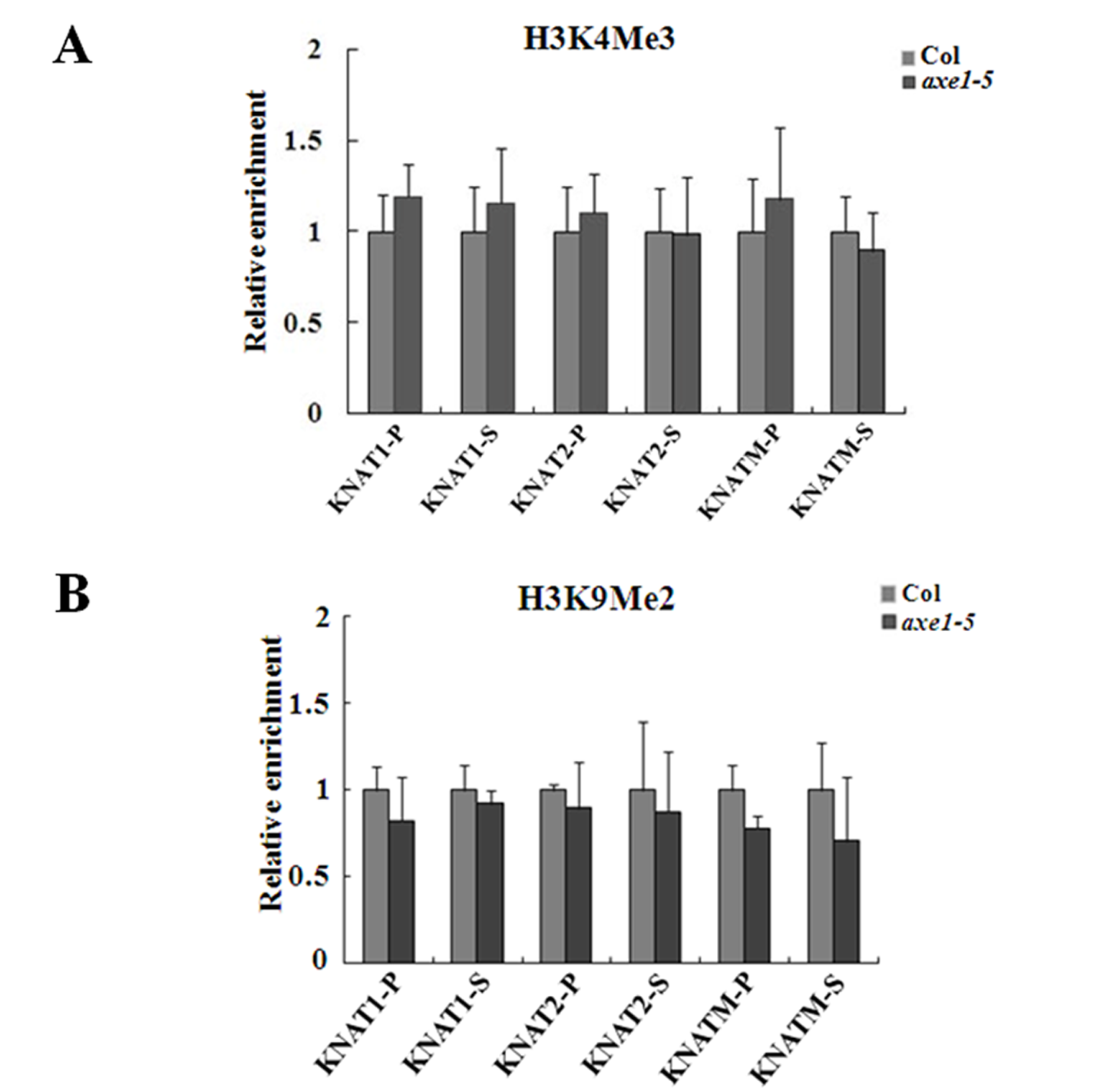

Supplement: Figure S6 — Levels of H3K4Me3 and H3K9Me2 in KNAT1, KNAT2 and KNATM in axe1-5 mutants. Relative levels of H3K4Me3 (A) and H3K9Me2 (B) in Col and axe1-5 mutant plants. P, promoter region; S, transcription start region. The amount of DNA after ChIP was quantified and normalized to an internal control (ACTIN2 or TA3). The values shown are means ± SD. (TIF) [file pgen.1003114.s006.tif]

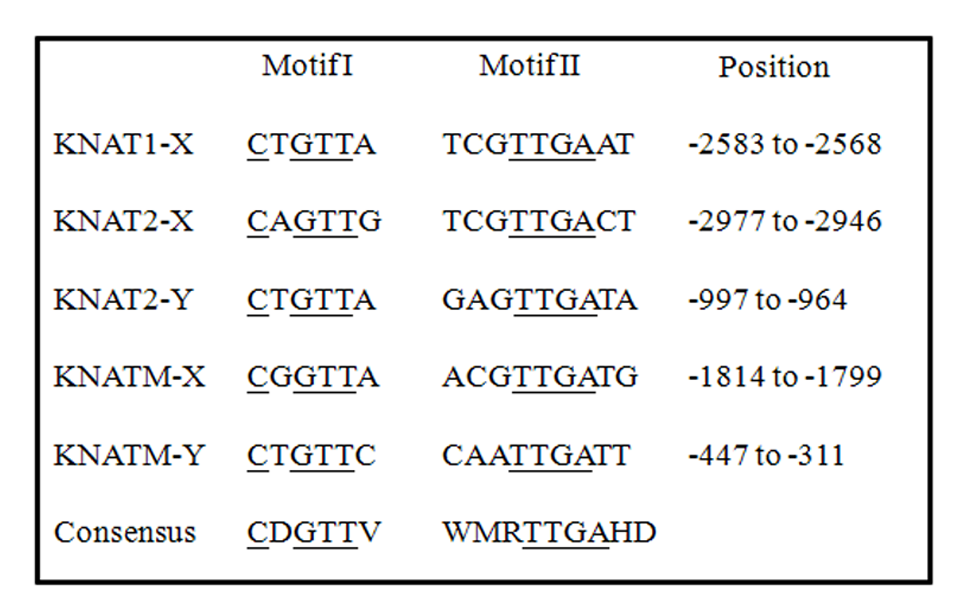

Supplement: Figure S7 — Sequences of motif I and motif II in KNAT1, KNAT2 and KNATM promoters. Inferred consensus sequences for the AS1 binding motifs and their positions relative to the translation start codon of KNAT1, KNAT2 and KNATM are also shown. (TIF) [file pgen.1003114.s007.tif]
