# Supplementary material for: Histone Deacetylase HDA6 Is Functionally Associated with AS1 in Repression of KNOX Genes in Arabidopsis
Source: PLoS Genet. 2012 Dec 13;8(12):e1003114. doi: 10.1371/journal.pgen.1003114 (PMC3521718; doi:10.1371/journal.pgen.1003114)
Supplement: Table S1 — Gene-specific primer pairs for quantitative RT–PCR. (DOC) [file pgen.1003114.s008.doc]

**Supplemental Table S1. Gene-specific primer pairs for quantitative RT-PCR**

| **Primers** | **Sequences 5'~3'** |
| --- | --- |
| UBQ1 | GATCTTTGCCGGAAAACAATTGGAGGATGGT |
| UBQ2 | CGACTTGTCATTAGAAAGAAAGAGATAACAGG |
| AS1-F | TAGAGGTCCATTGATCAGCC |
| AS1-R | TCCAACATCAGCTTCGTCAG |
| AS2-F | CTGAGTGATCTGTCCAATGG |
| AS2-R | CCACGGATTCTTGAATTGCC |
| KNAT1-F | GGGAAGAGTGACAATATGGG |
| KNAT1-R | TATGGACCGAGACGATAAGG |
| KNAT2-F | TCATCTGACGAGGAACTGAG |
| KNAT2-R | CGTCCATCATATCAATCGGC |
| KNATM-F | TGGGCTTCTTCTTCACTCAC |
| KNATM-R | TCGAGCAATACGCTTCCATG |
| CUC1-RT1 | CTGCAATTGCTCCGATCATC |
| CUC1-RT2 | GAGGCAGAGAAGGTAGATTC |
| CUC2-RT1 | TCCACTGTCCCTACTACTAC |
| CUC2-RT2 | TGACTCATTCTCTTCGGCAG |
| PHB-RT1 | TCTCAAACCAGTGGTGACAC |
| PHB-RT2 | AGAACTTTCCACACCGTTGC |
| PHV-RT1 | AGGTGCAGATCTGTTTGGAG |
| PHV-RT2 | TCGACACACATATTCCTGCC |
