# Supplementary material for: Histone Deacetylase HDA6 Is Functionally Associated with AS1 in Repression of KNOX Genes in Arabidopsis
Source: PLoS Genet. 2012 Dec 13;8(12):e1003114. doi: 10.1371/journal.pgen.1003114 (PMC3521718; doi:10.1371/journal.pgen.1003114)
Supplement: Table S2 — Primers used for quantitative RT–PCR analyses in ChIP assays. (DOC) [file pgen.1003114.s009.doc]

**Supplemental Table S2. Primers used for quantitative RT-PCR analysis in ChIP assays**

| **Primers** | **Sequences 5'~3'** |
| --- | --- |
| ACTIN2-F | CGTTTCGCTTTCCTTAGTGTTAGCT |
| ACTIN2-R | AGCGAACGGATCTAGAGACTCACCTTG |
| TUB2-F | ACAAACACAGAGAGGAGTGAGCA |
| TUB2-R | ACGCATCTTCGGTTGGATGAGTGA |
| TA3-F | GATTCTTACTGTAAAGAACATGGCATTGAGAGA |
| TA3-R | TCCAAATTTCCTGAGGTGCTTGTAACC |
| KNAT1-P-F | AACCATAGCCTGAAGTAGCC |
| KNAT1-P-R | AAGACGTCGTTTGCTTTGGG |
| KNAT1-S-F | CTCTTCATCTTACACCCATCC |
| KNAT1-S-R | CCAGGACCATAATTGCTAC |
| KNAT2-P-F | AACCGATCCGGTTAGACAAC |
| KNAT2-P-R | CTGTACGATTACATGGTTACG |
| KNAT2-S-F | CTTCAGAAGCAAGCACAAGG |
| KNAT2-S-R | TTCGTCGGATCCAAACAGTC |
| KNATM-P-F | GCACAAGTTTCATTGCCTTC |
| KNATM-P-R | AACCCTAGCTTCACACTTAG |
| KNATM-S-F | AAGGGTGGCTTGAGATTCTC |
| KNATM-S-R | TGGGCTTCTTCTTCACTCAC |
| KNAT1-X-F | TACACGAACACAGATGAT |
| KNAT1-X-R | CAGTGGAAGTGAGAGTAGG |
| KNAT1-Y-F | TAGATCCATATGGTTATGGGT |
| KNAT1-Y-R | CCTCTTATTTTCTGTTTCAGTA |
| KNAT2-X-F | CCTGAGCTAATTAAGTAGA |
| KNAT2-X-R | GGAGCTAATTTTGCTTATG |
| KNAT2-Y-F | CTGTCGTTTTTATAAGGTTTG |
| KNAT2-Y-R | CACTTATCGCACTTCTTGTTA |
| KNATM-X-F | AACCTTCTTCCTTCGAGCTC |
| KNATM-X-R | TATGGAAAGCACCGGGAAAC |
| KNATM-Y-F | GACAGTCTGACTCAAAGTGC |
| KNATM-Y-R | AACTCTGGTCGACATGTCAC |
